# Genome-wide identification and comparative evolutionary analysis of sorbitol metabolism pathway genes in four Rosaceae species and three model plants

**DOI:** 10.1186/s12870-022-03729-z

**Published:** 2022-07-15

**Authors:** Leiting Li, Meng Li, Juyou Wu, Hao Yin, Jim M. Dunwell, Shaoling Zhang

**Affiliations:** 1 College of Horticulture, Nanjing Agricultural University, Nanjing, Jiangsu China; 2 Shanghai Center for Plant Stress Biology and CAS Center for Excellence in Molecular Plant Sciences, Chinese Academy of Sciences, Shanghai, China; 3 School of Agriculture, Policy and Development, University of Reading, Earley Gate, Reading, UK

**Keywords:** Photosynthesis product, Rosaceae, Sorbitol-6-phosphate dehydrogenase (S6PDH), Sorbitol dehydrogenase (SDH), Sorbitol transporter (SOT)

## Abstract

In contrast to most land plant species, sorbitol, instead of sucrose, is the major photosynthetic product in many Rosaceae species. It has been well illustrated that three key functional genes encoding sorbitol-6-phosphate dehydrogenase (*S6PDH*), sorbitol dehydrogenase (*SDH*), and sorbitol transporter (*SOT*), are mainly responsible for the synthesis, degradation and transportation of sorbitol. In this study, the genome-wide identification of *S6PDH*, *SDH* and *SOT* genes was conducted in four Rosaceae species, peach, mei, apple and pear, and showed the sorbitol bio-pathway to be dominant (named sorbitol present group, SPG); another three related species, including tomato, poplar and *Arabidopsis*, showed a non-sorbitol bio-pathway (named sorbitol absent group, SAG). To understand the evolutionary differences of the three important gene families between SAG and SPG, their corresponding gene duplication, evolutionary rate, codon bias and positive selection patterns have been analyzed and compared. The sorbitol pathway genes in SPG were found to be expanded through dispersed and tandem gene duplications. Branch-specific model analyses revealed *SDH* and *S6PDH* clade A were under stronger purifying selection in SPG. A higher frequency of optimal codons was found in *S6PDH* and *SDH* than that of *SOT* in SPG, confirming the purifying selection effect on them. In addition, branch-site model analyses revealed *SOT* genes were under positive selection in SPG. Expression analyses showed diverse expression patterns of sorbitol-related genes. Overall, these findings provide new insights in the evolutionary characteristics for the three key sorbitol metabolism-related gene families in Rosaceae and other non-sorbitol dominant pathway species.

**Supplementary Information:**

The online version contains supplementary material available at 10.1186/s12870-022-03729-z.

## Introduction

Sugars, are important compounds in all plants, and play critical roles in both primary and secondary metabolism. Notably, sugars are important in Rosaceae fruit trees, where they accumulate in the fruits, which are the main organs consumed. Such fruit trees, which represent the most important sources of fruit for human consumption, include pear, apple, peach, and mei. Overall, the sugar content in fruit is determined by the carbon partitioning system in plants, a critical process that distributes chemical energy converted by the plant through photosynthesis [1]. Compared with most land plants, the source-sink system in pear and several other Rosaceae species, is different in terms of the type of sugar translocated from source to sink, which was sucrose in the former and sorbitol in the latter [2–4]. System evolution analyses of Rosaceae species showed that sorbitol is present in the Spiraeoideae and Dryadoideae subfamilies, whereas it is absent in the Rosoideae subfamily [5]. The soluble sugars in mature pear fruit comprise fructose, glucose, sucrose and sorbitol [6]. Sorbitol is not only a key metabolite in carbohydrate metabolism, but also a regulatory signal in stamen development, pollen tube growth and resistance response [7–9].

**Fig. 1:**
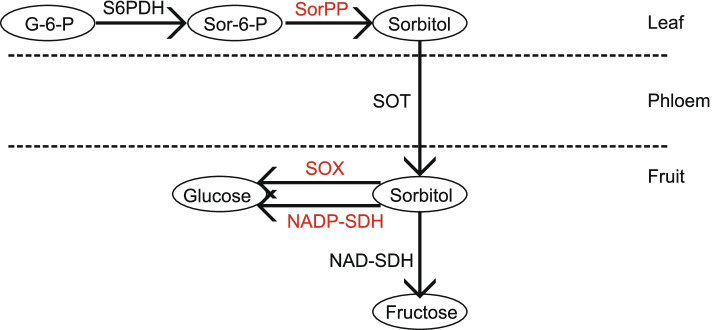
The scheme of sorbitol metabolism pathway for *S6PDH*, *SOT* and *SDH*. Note: SorPP, SOX and NADP-SDH are marked in red as their sequences have not been characterized in plants. G-6-P: glucose-6-phosphate, Sor-6-P: sorbitol-6-phosphate

The biosynthesis of sorbitol occurs in the cytosol of leaf cells and is different from sucrose in being produced from glucose 6-phosphate in two catalytic steps (Fig. 1). First, NADP-dependent sorbitol-6-phosphate dehydrogenase (S6PDH, EC 1.1.1.200) catalyzes glucose 6-phosphate into sorbitol 6-phosphate [10]. Secondly, sorbitol-6-phosphate phosphatase (SorPP, EC 3.1.3.50) catalyzes sorbitol-6-phosphate into sorbitol [11]. The transportation of both sucrose and sorbitol occurs from leaves to fruits through the phloem, but with different transporters, namely sucrose transporter (SUT) and sorbitol transporter (SOT). In fruits, sorbitol is converted into glucose or fructose by the activity of three enzymes, NADH-dependent sorbitol dehydrogenase (NAD-SDH, EC 1.1.1.14) [12], DADPH-dependent sorbitol dehydrogenase (NADP-SDH, EC 1.1.1.21) [13], and sorbitol oxidase (SOX, EC 1.1.3.x) [14]. In total, there are five enzymes and one transporter that are closely related to the biosynthesis, degradation and transportation of sorbitol in plants. Till now, three key genes, *S6PDH*, *SDH* and *SOT*, which are known to be involved in sorbitol biosynthesis, degradation and transportation, have been well demonstrated in plants [2].

*S6PDH*, localized mainly in leaf cytosol and chloroplast, has been reported to play multiple roles in plants, including not only cold, dark, and abscisic acid stresses [15], but also osmotic [16] and salt stresses [17]. SDH, a cytosolic protein required for sorbitol metabolism [18], emerged very early during evolution [19], and plays a role in abiotic stress in Arabidopsis [20] and tomato [21]. For example, overexpression of SDH in Arabidopsis confers tolerance to salt and osmotic stress [22]. In addition, the presence of SOT is correlated with the accumulation of sorbitol under conditions of drought stress in apple [23] and regulates sorbitol accumulation in pear fruit [24, 25]. Also, previous research has shown that *S6PDH*, *SDH* and *SOT* are members of larger gene families in apple [26], pear [27] and peach (*Prunus persica*) [28] genomes than in other plant genomes. This finding may be due gene duplication, which is an important feature of genome evolution [29].

Therefore, the aim of this study is to reveal the differences between contrasting species, in which the sugar pathway is either dominated by sorbitol or lacks sorbitol, by investigating the key genes *S6PDH*, *SDH* and *SOT*. Since the sugar pathway of pear (*Pyrus bretschneideri*), apple (*Malus domestica*), peach (*Prunus persica*) and mei (*Prunus mume*) in Rosaceae is dominated by sorbitol, in this study, we designated these species as the sorbitol present group (SPG) group. In contrast, *Arabidopsis*, a model eudicot plant, poplar (*Populus trichocarpa*), a model woody plant species, and tomato (*Solanum lycopersicum*), a model fruit plant, are members of the sorbitol absent group (SAG) group. We identified *S6PDH*, *SDH* and *SOT* genes of the SAG and SPG groups through a cluster of orthologous groups of proteins (COG) method, then created phylogenetic trees for them and performed evolutionary rate and codon usage bias analyses. We compared the evolutionary pattern of different genes in the two groups to determine their individual features. Overall, our study was designed to provide new insights into the evolutionary characteristics for the three key sorbitol metabolism-related gene families in the Rosaceae and other non-sorbitol dominant pathway species.

## Materials and methods

### Genome resources

**Fig. 2:**
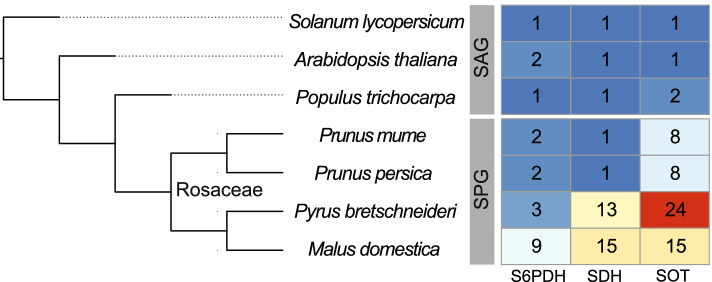
Phylogenetic tree for seven species used in this study. Note: Right table showed the number of genes

Evolutionary analysis was conducted on seven species based on whether sorbitol was present as the major translocated sugar. These species were designated as either the sorbitol present group (SPG) or the sorbitol absent group (SAG). The SPG includes pear (*Pyrus bretschneideri*), apple (*Malus domestica*), peach (*Prunus persica*) and mei (*Prunus mume*) in Rosaceae and the SAG includes *Arabidopsis*, poplar (*Populus trichocarpa*), and tomato (*Solanum lycopersicum*). The phylogenetic tree of these seven species is shown in Fig. 2. Genome resources of the various species were collected from public databases (Table 1). The apple, peach, tomato, poplar, and *Arabidopsis* genome sequences were retrieved from Phytozome version 9 (http://www.phytozome.net). The pear genome sequences were retrieved from GigaDB (http://gigadb.org/dataset/100083) and the mei genome sequences were retrieved from the Mei Genome Project website (https://github.com/lileiting/prunusmumegenome).

**Table 1:** Evolutionary rate analyses of *S6PDH*, *SDH* and *SOT* gene families using branch-specific model of PAML

Gene	Model	ω setting	-*ln* L	Estimated parameters	Likelihood ratio test
S6PDH	One-ratio	Entire tree: ω_0_	6724.98	ω_0_ = 0.164	
	Two-ratio	branch A & B: ω_1_	6718.04	ω_1_ = 0.515	two-ratio vs. one-ratio: *P* < 0.01
		other branches: ω_0_		ω_0_ = 0.148	
	Three-ratio	Branch A: ω_1_	6714.01	ω_1_ = 0.043	Three-ratio vs. one-ratio: *P* < 0.01
		Branch B: ω_2_		ω_2_ = 0.629	
		Other branches: ω_0_		ω_0_ = 0.152	
SDH	One-ratio	Entire tree: ω_0_	9280.82	ω_0_ = 0.104	
	Two-ratio	branch A: ω_1_	9278.32	ω_1_ = 0.211	two-ratio vs. one-ratio: *P* = 0.03
		other branches: ω_0_		ω_0_ = 0.101	
SOT	One-ratio	Entire tree: ω_0_	28,847.56	ω_0_ = 0.192	
	two-ratio	branch A&B&C: ω_1_	28,841.07	ω_1_ = 0.412	two-ratio vs. one-ratio: *P* < 0.01
		other branches: ω_0_		ω_0_ = 0.186	
	Four-ratio	Branch A: ω_1_	28,840.62	ω_1_ = 0.346	Four-ratio vs. one-ratio: *P* < 0.01
		Branch B: ω_2_		ω_2_ = 0.374	
		Branch C: ω_3_		ω_3_ = 0.581	
		Other branches: ω_0_		ω_0_ = 0.186	

ω_0_ means the overall evolutionary ratio for one-ratio model and background evolutionary ratio for two-ratio or three-ratio models

ω_1_,ω_2,_ω_3_ indicates evolutionary ratio for branches indicated

### Gene identification

Protein sequences from the selected genomes were first used to build a cluster of orthologous groups (COG) dataset [30–32]. This procedure was based on the concept that a family of in-paralogs in one lineage can be orthologous to a single gene in another lineage and orthologs could be identified by the identification of an orthologous family. In such a family, a pair of sequences could be identified as two kinds of relationship, i.e. symmetrical and asymmetrical BeTs (the Best Hits). The orthologous family would form a network after linking the symmetrical and asymmetrical BeTs by solid and broken lines; and thus all the members in the network could be identified with one member investigated [30, 31, 33, 34]. Briefly, there were five steps. First, two types of all-against-all protein sequence comparisons were carried out using PSI-BLAST with and without the SEG filter (low complexity masking), and composition-based score adjustment [35] was carried out. Both methods used the parameter of “-show_gis -outfmt 7 -num_descriptions 1000 -num_alignments 1 000 -dbsize 100000000” as suggested [34]. Secondly, after processing the BLAST results with an E-value threshold of 0.1 using the program COGreadblast, and collecting lineage-specific expansion using the program COGlse, clusters were made from symmetrical best hits using the program COG triangle with an E-value threshold of 0.01 and a hit coverage threshold of 0.5 [34]. Thirdly, three representative proteins of S6PDH, SDH and SOT with status of reviewed were retrieved from the Uniprot database (http://www.uniprot.org/) with accession number of P28475, Q9FJ95, Q8RI1 (Table S1). Fourth, each corresponding locus was queried against COG datasets constructed from the second step and the respective COGs were obtained. Fifth, each sequence in the obtained COG was repeated as in the fourth step, until no new sequence was found. The presence of a specific gene in multiple COGs was allowed, in order to ensure all homologs were included. To further confirm the results, the identified genes were submitted to CDD [36] to determine their protein domains. Results from CDD confirmed their membership of superfamilies; specifically, all *S6PDH* genes belonged to the aldo-keto reductase (AKR) superfamily (cl00470), all *SDH* genes belonged to the medium-chain dehydrogenases/reductases (MDR) superfamily (cl16912), and all *SOT* genes belonged to the major facilitator superfamily (MFS, cl21472).

### Phylogenetic analysis

The coding sequences of genes in each gene family were aligned using the codon model in PRANK [37]. The aligned sequence was then translated into amino acids and the best substitution models were tested using Prottest 3 [38]. The results showed that for *S6PDH*, the best evolutionary model implemented in RAxML [39] is LG + G (the substitution matrix [40], and a gamma model of rate heterogeneity), and the best evolutionary model implemented in MrBayes [41] is JTT + G (the substitution matrix [42], and a gamma model of rate heterogeneity). For *SDH*, the best evolutionary model implemented in RAxML and MrBayes were both JTT + G. For *SOT*, the two best evolutionary models implemented in RAxML and MrBayes were JTT + I + G (the substitution matrix [42], a proportion of invariant sites, and a gamma model of rate heterogeneity).

Phylogenetic trees were constructed using the Bayesian method implemented in MrBayes [41], running one million generations for each gene family, and discarding the first 25% samples as burn-in. Convergence was assessed by the potential scale reduction factor [43]. Additionally, maximum likelihood trees with 1000 bootstrap replicates using RAxML [39] were reconstructed and bootstrap support values were added to the Baysian trees using SumTrees [44]. Branches in the phylogenetic trees with posterior probabilities less than 0.80 were removed using Dendroscope 3 [45] and phylogenetic trees were visualized using FigTree (http://tree.bio.ed.ac.uk/software/figtree/). Genes in the SPG were divided into different clades by the distribution of genes in combination with the species tree. Each clade in a gene family typically contains genes from the four SPG species and clades in the same gene family share the common ancestor corresponding to the speciation of SPG species.

### Gene duplication type identification

Protein sequences for each of the seven species were independently performed with an all-against-all BLASTP search with E-value threshold of 1e-5 to search for potential anchors between every possible pair of chromosomes. The homologous genes were used as input for the program MCScanX [46] to search collinear blocks and gene types. Four types of gene duplication including dispersed, proximal, tandem and WGD/segmental were assigned by MCScanX.

### Pairwise K_a_/K_s_ calculation

The coding sequences of each group of genes was pairwise aligned using PRANK [37], and then the alignment sequences to AXT format were converted and imported into KaKs_Calculator 2.0 [47] to calculate K_a_/K_s_ using YN model. The R programing language (http://www.r-project.org) was used to make the boxplot.

### Estimation of branch-specific evolutionary rates and detection of positive selection

The coding sequences of each gene family were aligned using a codon model in PRANK [37]. The evolutionary rate of each gene family and branches for SPG, was estimated using branch-specific model in PAML 4.7a [48]. First, we used a one-ratio model, assuming the evolutionary rate in the whole phylogenetic tree was the same. Secondly, we used a two-ratio model, assuming the evolutionary rate in the SPG genes is different from the SAG genes. Thirdly, we used a multiple-ratio model, assuming each branch representing the speciation of sorbitol present species was independent, and has a different ratio from background ratio. Furthermore, a likelihood ratio test (LRT) was used to test if the two-ratio model rejects the one-ratio model, or the multiple-ratio model rejects the one-ratio models.

To test if those clades of SPG, as marked in Fig. 2 for the three gene families, were subject to positive selection, we used the program Fitmodel [49] to conduct the selection analysis, which was a maximum likelihood-based program used for estimating parameters of sequence evolution. Fitmodel allowed the site-specific selection process to vary along lineages for a phylogenetic tree (switching model). M3 [50] and M3 + 1 [49] models in Fitmodel were employed in this analysis to test positive selection sites.

### Codon usage bias analysis

The overall codon bias for all genes in the seven genomes was calculated using the method of effective number of codons (ENC) [51] with the ENCprime package [52]. The measure does not make any assumptions, including optimal codons or GC contents. Values of Nc ranged from 20, for extremely biased genes that use only one codon per amino acid, to 61, for genes that use all synonymous codons equally [51, 53]. Short sequences of less than 50 codons were removed from the analysis. The optimal codon for seven species was determined using a method similar to that of [53]. Briefly, the correlation of codon frequency of each codon in their codon family was calculated with the overall codon bias (Nc). The optimal codon for each codon family was defined as t2he codon that showed the strongest and most significant negative correlation with Nc. Codon families that appeared less than 10 times were removed. The threshold of significance is 0.05/n, where n is the number of codons in the codon family. Spearman correlation was performed using the R programming language.

Frequency of optimal codons (FOP) was defined as the ratio of optimal codons to the sum of non-optimal codons and optimal codons. The formula is as follows: FOP = number of optimal codons / (Number of optimal codons + Number of non-optimal codons). In addition, GC content and GC3 content were calculated using CodonW package (http://codonw.sourceforge.net).

### Expression analysis

In total, 20 libraries for apple, mei, pear and peach were retrieved from the SRA database (http://www.ncbi.nlm.nih.gov/sra) and used to perform expression analysis for *S6PDH*, *SDH* and *SOT* genes.

These included five libraries for apple, comprising one leaf library (SRR767660) and four fruit libraries from different developmental stages, 25 daa (days after anthesis), 35 daa, 60 daa and 87 daa (SRR768128, SRR768129, SRR768130, SRR768131); five libraries for mei, comprising bud (SRR542478), leaf (SRR542479), root (SRR542480), stem (SRR542481) and fruit (SRR542482); six libraries for pear fruit (SRR654690, SRR654692, SRR654693, SRR654695, SRR654699, SRR654700); and four libraries for peach, comprising leaf (SRR531862), root (SRR531863), fruit (SRR531864), and embryos and cotyledons (SRR531865).

The downloaded SRA format data were first converted to FASTQ format using the SRA toolkit (https://trace.ncbi.nlm.nih.gov/Traces/sra/sra.cgi?view=software), then mapped to reference genomes with Tophat v2.1.0 [54] and normalized to fragments per kilobase exons per million reads (FPKM) using Cufflinks v2.2.1 [55] with default parameters. The expression data were subjected to log2 transformation and then visualized using MeV (http://www.tm4.org/mev.html).

## Results

### Expansion in size of *S6PDH*, *SDH* and *SOT* gene families

The expansion of gene family size (typically by gene duplication) is important for biological evolution by supplying greater genetic diversity (Zhang 2003). To investigate differences in gene family size between the SPG (peach, mei, pear and apple) and the SAG (tomato, Arabidopsis, and poplar), the identified genes in the *S6PDH*, *SDH* and *SOT* families were compared. In total, 20 *S6PDH*, 33 *SDH* and 59 *SOT* genes were identified (Fig. 2, Table S2). In these gene families, the average gene numbers in SPG (4.0 for *S6PDH*, 7.5 for *SDH* and 13.75 for *SOT*) are larger than those in the SAG (1.3 for S6PDH, 1.0 for SDH and 1.3 for SOT), indicating gene family size expansion is contributing to the evolution of the sorbitol character. Although the gene numbers in pear and apple are constantly larger than those in the SAG, this is not the case for the *S6PDH* and *SDH* genes in peach and mei, which have similar or identical gene numbers, that is one or two. This indicated that gene family size expansion did not necessarily exist in all three gene families. Only the *SOT* family was expanded in peach and mei. The expansion in gene family size in pear and apple (Fig. 2) could be partially explained by recent whole genome duplication [26, 27]; for example, the number of SOT genes in the apple genome (15 genes) is about twice that in peach and mei genomes (both have 8 genes), which have no recent whole genome duplications [28, 56].

### Distinct evolutionary divergence pattern for *S6PDH*, *SDH* and *SOT* gene families

**Fig. 3:**
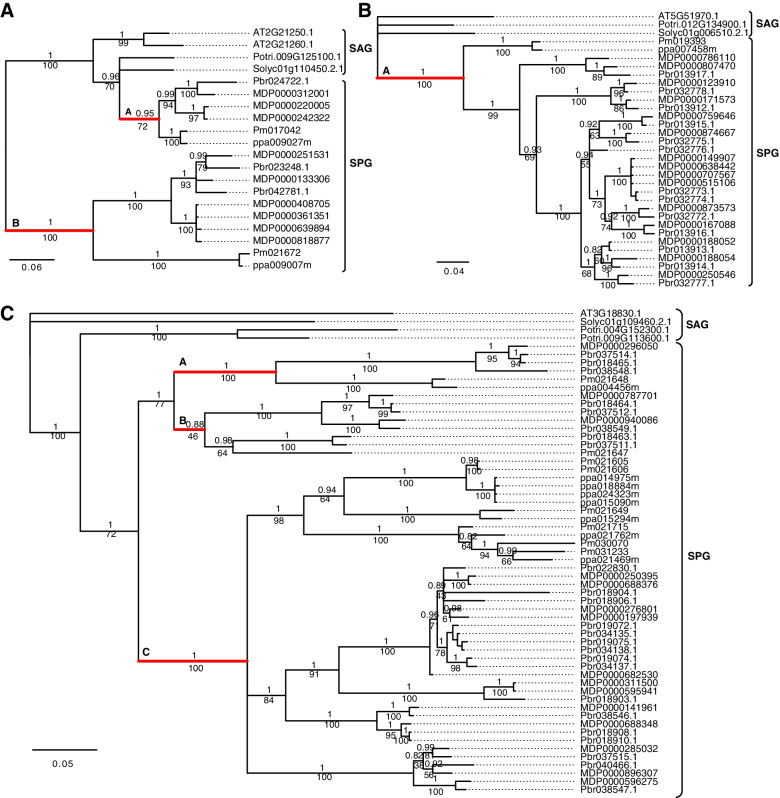
Phylogenetic tree of *S6PDH*, *SDH* and *SOT* gene families. **A** *S6PDH* gene family, **B** *SDH* gene family, **C** *SOT* gene family. Note: Posterior probability is labeled in the upper side of each branch and bootstrap support value is labeled in the side of each branch. The gene duplication type is labeled for each gene

To investigate the phylogenetic relationships of the *S6PDH*, *SDH* and *SOT* gene families in the seven species, we constructed phylogenetic trees (Fig. 3) using Bayesian and maximum likelihood methods, which showed different evolutionary patterns. We found that genes in the SPG were divided into different numbers of clades. Each clade contains genes from four SPG species and different clades of the same gene family share the common ancestor in the ancestral genome of the four SPG species. There were two clades for *S6PDH* (Fig. 3A), one clade for *SDH* (Fig. 3B) and three clades for *SOT* (Fig. 3C) in the SPG. This indicated that the *S6PDH* and *SOT* gene families were duplicated, and corresponded with the generation of sorbitol characteristics in Rosaceae. In addition, the number of genes of different clades was uneven with more genes in the *S6PDH* clade B (10) than clade A (6) (Fig. 3A), and more genes in the *SOT* clade C (41) than in clades A (6) and B (8) (Fig. 3C). Such clades with more genes may be a consequence of the greater number of gene duplication events. To further illustrate how those genes increased in number, intra-genome synteny analysis (Table S3) was performed. The results showed evidence of distinct gene family expansion. For *S6PDH*, 13 out of 20 genes were dispersed and duplicated; for *SDH*, 10 and 11 genes out of 33 *SDH* genes were dispersed and tandem duplicated; for *SOT*, 29 and 17 out of 59 *SOT* genes were dispersed and tandem duplicated, respectively.

### Purifying and positive selection in *S6PDH*, *SDH* and *SOT* gene families

**Fig. 4:**
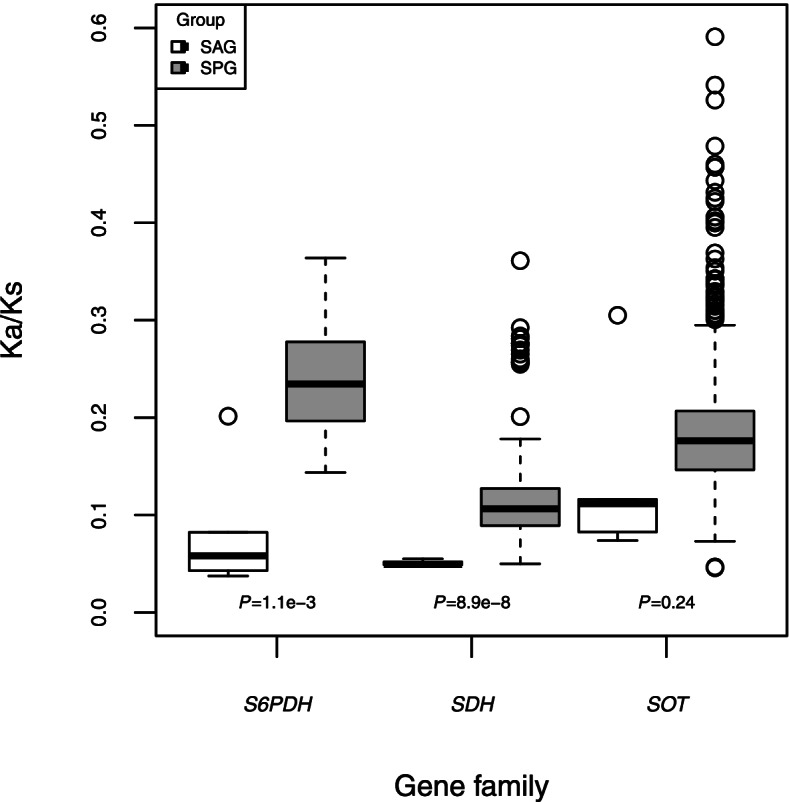
Comparison of pairwise K_a_/K_s_ values between SAG and SPG for *S6PDH*, *SDH* and *SOT* gene families. Note: sorbitol present group (SPG) and sorbitol absent group (SAG)

To test the selection pressure for *S6PDH*, *SDH* and *SOT* gene families, we performed both branch-specific models of PAML and pairwise Ka/Ks analyses (Fig. 4). For the branch-specific model analyses, we used one-ratio, two-ratio and multiple ratio models (Table 2). The one-ratio model assumed genes on the phylogenetic tree had the same evolutionary ratio, and the results showed that *SOT* (ω_0_ = 0.192) had the greatest evolutionary ratio, *SDH* had the smallest evolutionary ratio (ω_0_ = 0.104), and evolutionary ratio for *S6PDH* (ω_0_ = 0.164) was intermediate. The two-ratio model assumed genes in SPG had the same evolutionary ratio (ω_1_, foreground evolutionary ratio) but had a different background ratio (ω_0_). The results showed a higher foreground evolutionary ratio for three gene families, among which ω_1_ = 0.515 for *S6PDH*, ω_1_ = 0.211 for *SDH*, and ω_1_ = 0.412 for *SOT*. All the genes in our study have been through a purifying selection and *SDH* underwent the strongest purifying selection. In contrast, purifying selection was relaxed in the *S6PDH* and *SOT* genes in the SPG. To confirm the results, we performed pairwise Ka/Ks analyses for genes of each gene family in SAG and SPG (Fig. 4). The results showed that the average Ka/Ks value of *SDH* (average Ka/Ks = 0.113) was smaller than that of *S6PDH* (average Ka/Ks = 0.241) and *SOT* (average Ka/Ks = 0.181) in the SPG group. Similarly, stronger purifying selection was observed in the SPG genes. As *S6PDH* and *SOT* genes in the SPG have a complex evolutionary history with more than one clade, the multiple-ratio model for branch-specific model analyses was applied to test selection pressure in each clade in *S6PDH* and *SOT*. Interestingly, inside the SPG clades, two clades of *S6PDH* have a very different evolutionary ratio. Clade B had a value (ω_2_ = 0.629) greater than the background ratio (ω_0_ = 0.152), while clade A (ω_1_ = 0.043) was less than the background ratio, which indicated an even stronger purifying selection of *S6PDH* clade A. The *SOT* clade C (ω_3_ = 0.581) had a higher evolutionary ratio than *SOT* clade A (ω_1_ = 0.346) and *SOT* clade B (ω_2_ = 0.374) (Table 2), indicating the *SOT* clade C has undergone relaxed purifying selection like *S6PDH* clade B. Furthermore, a branch-site model was used to investigate whether the three gene families have positive selection after the divergence of sorbitol present species. We first ran M3 and M3 + 1 models in Fitmodel for each branch, and examined them using a likelihood ratio test. The results showed that all the three branches in *SOT* showed significance by likelihood ratio test (LRT), indicating *SOT* genes were positively selected in the identified sites. In total, 44 sites in *SOT* branch A, 8 sites in *SOT* branch B, and 8 sites in *SOT* branch C were positively selected.

**Table 2:** Statistics summary for detecting positive selection using M3 and M3 + 1 model of Fitmodel

Gene family	Branch	M3 model		M3 + 1 model		Positive sites (M3 model)
		-ln L	*p*_1_ *p*_2_ *p*_3_	-ln L	*p*_1_ *p*_2_ *p*_3_	
			*ω*_**1**_ *ω*_**2**_ *ω*_**3**_		*ω*_**1**_ *ω*_**2**_ *ω*_**3**_	
S6PDH	A	2451.93	0.95 0.05 0.00	2451.89	0.96 0.00 0.04	–
			0.167 2.610 2.610		0.165 3.134 3.158	
	B	2830.82	0.50 0.39 0.11	2830.29	0.61 0.00 0.38	–
			0.000 0.759 0.784		0.000 1.004 1.014	
SDH	A	6899.7	0.77 0.14 0.09	6895.26	0.83 0.06 0.11	–
			0.040 0.305 0.601		0.030 0.426 0.751	
SOT	A	4560.88	0.59 0.09 0.32	4552.25^**^	0.88 0.01 0.11	168, 228, 234, 238, 247, 270, 277, 286, 306, 317, 335, 342, 343, 362, 392, 397, 401, 416, 425, 426, 449, 453, 461, 477, 482, 484, 485, 489, 506, 526, 543, 544, 550, 555, 556, 593, 598, 602, 628, 657, 664, 667, 671, 690
			0.020 0.022 0.828		0.000 0.001 2.830	
	B	5125.58	0.59 0.28 0.13	5118.77^**^	0.78 0.20 0.03	14, 331, 392, 436, 579, 589, 592, 600
			0.035 0.258 1.277		0.016 0.755 5.594	
	C	15,493.35	0.49 0.38 0.12	15,473.31^**^	0.60 0.29 0.11	674, 710, 1002, 1177, 1191, 1204, 1275, 1346
			0.034 0.332 0.956		0.008 0.457 1.258	

*p*_i_ proportion of sites that fall into *ω*_**1**_ site class, i = 1, 2, 3

* *p* < 0.05

** *p* < 0.01

### Codon bias pattern of *SDH* is different from *S6PDH* and *SOT*

**Fig. 5:**
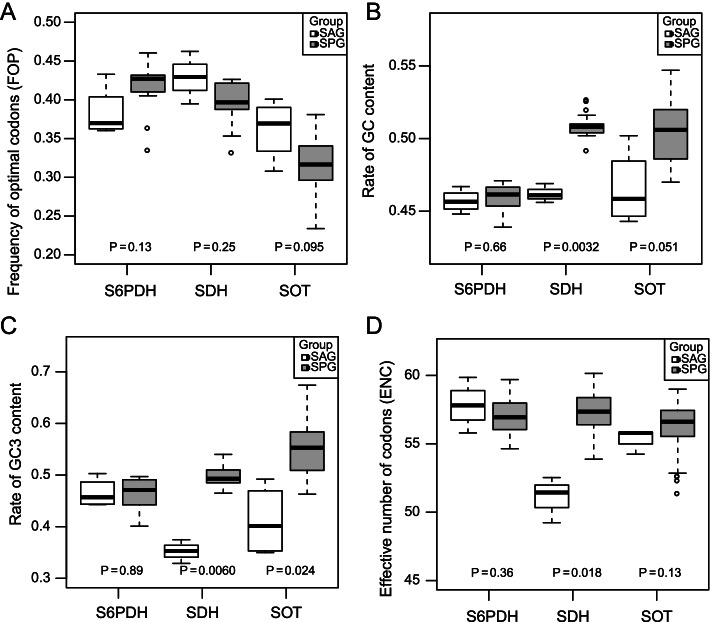
Codon bias pattern analysis between SAG and SPG for *S6PDH*, *SDH* and *SOT* gene families. **A** Frequency of optimal codons (FOP); **B** effective number of codon (ENC); **C** Rate of GC content; **D** Rate of GC3 content

Levels of codon bias are often used as an indicator of the efficiency of purifying selection [57, 58]. To determine the strength of such strong purifying selection in *SDH* identified above, frequency of optimal codons (FOP) was calculated to explore codon bias in the SAG and SPG genes of three gene families (Fig. 5A and Table S4). Increased FOP indicates increased codon bias. The results showed that, in SAG, *SDH* genes have the highest average value of FOP, which was consistent with the strongest purifying selection as mentioned in the previous section. The FOP of *SOT* in both SAG and SGP genes was lowest, which was consistent with the relaxation of purifying selection observed in *SOT*. As FOP may be related to GC or GC3 bias [58], we also measured the GC and GC3 contents (Fig. 5B and C). The results showed that GC and GC3 contents increased from significantly lower than 50% to around 50% for *SDH* and *SOT* in SPG than SAG. This indicated that the relaxation of purifying selection of *SDH* and *SOT* in SPG was largely driven by the flexibility of GC and GC3 contents. Since ENC was not affected by the GC and GC3 contents in the same manner as FOP [58], we also measured the ENC values (Fig. 5D). The results also showed the strongest codon bias of *SDH* in the SAG (by lowest ENC), confirming the strong purifying selection in *SDH*, irrespective of GC and GC3 bias.

### Tissue-specific expression profiles of *S6PDH*, *SDH* and *SOT* genes in SPG species

**Fig. 6:**
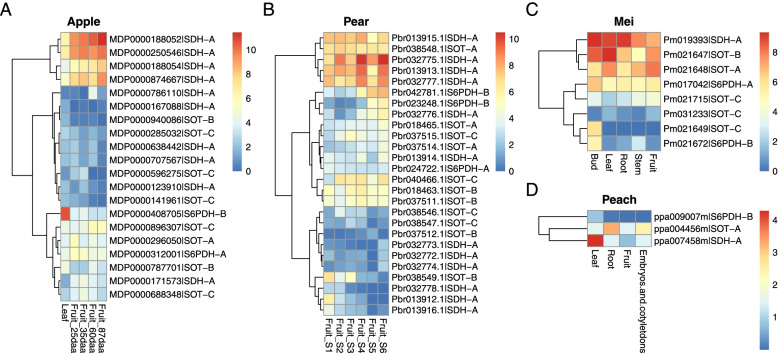
Expression profiles of *S6PDH*, *SDH* and *SOT* genes in different tissues of apple, pear, mei and peach. **A** The leaf tissue and four developmental stages of fruits of apple (25 daa (days after anthesis), 35 daa, 60 daa, and 80 daa). **B** The pear fruit in six developmental stages (S1: 15 days after flowering; S2: 36 days after flowering; S3: 80 days after flowering; S4: 110 days after flowering; S5: 145 days after flowering; S6: 167 days after flowering). **C** The bud, leaf, root, stem, and fruit tissues of mei. **D** The leaf, root, fruit, and embryo and cotyledon tissues of peach. Note: The expression data were normalized using fragments per kilobase of exon per million mapped reads (FPKM), with log2 transformed

To examine the expression pattern of *S6PDH*, *SDH* and *SOT* genes in the four SPG species, we collected and analyzed RNA-seq data from public databases (Fig. 6). The results showed that only a proportion of the genes were expressed in the examined samples and genes were expressed differently in different tissues. Apple genes were examined in one leaf and four fruit tissues (25 days after anthesis (daa), 35 daa, 60 daa, and 87 daa) of ‘Golden Delicious’. The results showed that there were 2 *S6PDH*, 10 *SDH*, and 8 *SOT* genes expressed (FPKM > 0.5) in these tissues (Fig. 6A). Among these genes, MDP0000408705 in *S6PDH* clade B was specifically highly expressed (FPKM > 1000) in leaf. In addition, some other genes like MDP0000312001 (*S6PDH*-A), MDP0000250546 (*SDH*-A) and MDP0000787701 (*SOT*-B) were also highly expressed in the leaf tissue, indicating their important roles in sorbitol metabolism in this tissue, particularly the accumulation of sugar compounds. Expression levels of four *SDH* genes (MDP0000188052, MDP0000250546, MDP0000188054, MDP0000874667) were found to be correlated with apple fruit maturation, with increasing expression at four time points of fruit development. Interestingly, the former three of the *SDH* genes were located in the same sub-clade in the phylogenetic tree (Fig. 3B), indicating coding sequence pattern may play a role in the expression pattern. Additionally, the *SOT* genes showed temporal expression divergence; some were highly expressed in the first stage of fruit development (like MDP0000296050) and some were highly expressed in the later stage (like MDP0000896307). Pear genes showed similar patterns, in that some of the genes (3 *S6PDH*, 12 *SDH*, and 11 *SOT*) were expressed (FPKM > 0.5) at six stages (S1: 15 days after flowering (daf); S2: 36 daf; S3: 80 daf; S4: 110 daf; S5: 145 daf; S6: 167 daf) of fruit development (Fig. 6B). Two of the *S6PDH* genes (Pbr042781.1, Pbr023248.1) tended to be expressed in the later stage of fruit development. Some of the *SDH* genes were highly expressed in almost all fruit developmental stages (Pbr032775.1, Pbr013913.1), some were highly expressed in the early stages only (Pbr013912.1), and some were highly expressed in the later stages (Pbr032776.1), indicating temporal expression divergence in this gene family. In the mei genome, 2 *S6PDH*, 1 *SDH*, and 5 *SOT* genes were found to be expressed in five tissues (bud, leaf, root, stem, and fruit) (Fig. 6C). Among these, Pm017042 (S6PDH-A), Pm019393 (SDH-A), Pm021648 (SOT-A), and Pm021647 (SOT-B) were more highly expressed in the leaf tissue, indicating their important roles in sorbitol accumulation. Additionally, only three genes from the peach genome were found to be expressed in the four tissues (leaf, root, fruit, and embryos and cotyledons), one for each gene family (Fig. 6D).

## Discussion

To understand how genes related to sorbitol have evolved in the context of the major photosynthetic product changing from sucrose in most land plants to sorbitol in many species in the Rosaceae, this study first performed comparative analyses of three key genes related to sorbitol metabolism (*S6PDH* for biosynthesis, *SDH* for sorbitol degradation, and *SOT* for sorbitol transportation) in two groups of species, with and without sorbitol as the major photosynthetic product. Although there were some previously published studies related to *SDH* [59] and *SOT* [60] genes, they were only focused on either one gene family or one species. Compared with these two previous studies, we found that the number of genes identified in the present study is similar, with minor differences due to identification methodology. The seven species used in the present study were also involved in a previous study that identified *SDH* genes from 42 angiosperm species [59]; the previous results were similar to those from the present study. However, gene number for five of these species were different, i.e. 2, 3, 4, 16, and 5 genes for poplar, mei, peach, apple and pear in the previous study, but they were 1, 1, 1, 15 and 13 in this study. This was mainly due to the fact that [59] designated L-idonate-5-dehydrogenase (LIDH, EC 1.1.1.264) as *SDH* Class II and included it in the analyses, while in this study, the *LIDH* genes were not included since they are not present in the SAG group. Additionally, *SDH* genes for pear were identified from the literature in [59], rather than from the pear genome resource as in this study. The *SOT* genes were designated as polyol/monosaccharide transporter (PLT) in the previous study for sugar transporter genes in pear [60], which identified 23 PLT/SOT genes. In this study, the number to 24 *SOT* genes with the addition of Pbr018904.1. To examine the putative function of Pbr018904.1, we performed a homology search against public databases using NCBI BLAST [61] and confirmed this gene as *PLT/SOT*.

The sorbitol characteristic was first identified in the Spiraeoideae and Dryadoideae subfamilies but not in the Rosoideae subfamily [5], indicating a stable genetic mechanism was present and had spread in the former subfamilies. Since *S6PDH*, *SDH* and *SOT* were key genes involved in sorbitol metabolism, it is likely that they have undergone evolutionary changes to generate this new character. In this study, we found *S6PDH*, *SDH* and *SOT* have evolved into two, one and three clades (Fig. 3), respectively in SPG. As the formation of clades corresponded to the speciation of SPG species, this led to the hypothesis that *S6PDH* and *SOT* are more relevant than *SDH* in the evolution of the sorbitol characteristic. The fact that only single *SDH* genes were identified in peach and mei (Fig. 2) supported this hypothesis. However, the surprising expansion of *SDH* genes in apple and pear caused a reconsideration of the evolution of *SDH* to the sorbitol characteristic, especially in apple and pear genomes. Although about half of the *SDH* in the pear genome were duplicated through WGD or segmental duplication (Fig. 2 and Table S3), such duplication type could not explain the expansion in the apple genome. If we take *SDH* class II [59] into account, we could reject the hypothesis that *S6PDH* and *SOT* are more relevant in the evolution of the sorbitol characteristic, because there were three peach and two mei genes in the *SDH* class II. Altogether, three gene families are important for the evolution of the sorbitol characteristic.

The evolution of plant traits may accompany selection pressure of related genes, either positive or purifying selection. We compared selection pressure in three gene families and confirmed the presence of strong purifying selection by codon bias analysis. Evolutionary rates are number of substitutions (fixed mutations) per unit of evolutionary time, which is typically estimated by the ratio ω = dN/dS (or Ka/Ks), where dN (Ks) indicates the non-synonymous substitution rate and dS (Ks) indicates the synonymous substitution rate [62]. The ratio ω is generally used as an indicator of positive selection (ω > 1) and purifying selection (ω < 1). Positive selection is the selection of beneficial alleles, while purifying selection is the selection against deleterious alleles [63]. The branch-specific model for the *S6PDH*, *SDH* and *SOT* gene families showed genes in SPG were found with evolutionary rates less than 1 (ω < 1), indicating purifying selection for all sorbitol metabolism pathway-related genes in this group. On the other hand, results for evolutionary rates for different branches with the multiple-ratio model indicated unbalanced evolutionary rates. The *S6PDH* clade A and *SDH* clade A were both found to be under strong purifying selection, while *S6PDH* clade B and *SOT* clade C both had with a relatively higher evolutionary rate, indicating reduced evolutionary constraints, which were driven by the result of relaxation of purifying selection [64]. This could be ascribed to the sorbitol character. Similarly, a higher evolutionary ratio was found to be associated with higher protein synthesis in dicots compared with monocots [30]. Although *SDH* Class II was not involved in this study, it was found to be positively selected [59]. Then, we estimated the codon bias to confirm the identified purifying selection. The results were confirmed by a relatively higher value of FOP in *S6PDH* and *SDH* genes in SPG. Different synonymous codons are favored by natural selection for translation efficiency and accuracy in different organisms [53]. Codon usage bias is not only a ubiquitous phenomenon observed in bacteria, plants and mammals [65, 66], but also plays a role as a means to fine-tune gene expression [67].

The duplicated members of sorbitol-related genes should lead to diversified gene expression patterns in the view of temporal and spatial differences. The expression analysis of *S6PDH*, *SDH* and *SOT* genes in the four SPG species supported the hypothesis that genes were differentially expressed in different tissues and different stages of fruit development.

## Conclusion

In this study, we first performed comparative evolutionary analyses for three keys genes (*S6PDH*, *SDH* and *SOT*) involved in the sorbitol metabolism pathway in two groups of species, with (SPG) and without (SAG) sorbitol as the major photosynthetic product. We found that the number of genes in the three gene families were expanded in SPG through dispersed and tandem duplication. *SDH* and *S6PDH* clade A in SPG were found to be under strong purifying selection. Branch-site model analysis revealed *SOT* genes in SPG were under positive selection. Codon usage revealed a higher frequency of optimal codons for *S6PDH* and *SDH* than *SOT* genes, confirming the effect of purifying selection. Expression analyses in fruit and leaf tissues identified important genes involved in sorbitol metabolism. Overall, this study provides further insights for understanding the underlying molecular mechanism for sorbitol metabolism.

## Supplementary Information

**Additional file 1: Supplementary Table 1.** Query sequences of S6PDH, SDH and SOT. **Supplementary Table 2.** List of identified genes for S6PDH, SDH, and SOT gene families. **Supplementary Table 3.** Information of identified genes for S6PDH, SDH, and SOT gene families. **Supplementary Table 4.** Optimal codons for seven species.

## Data Availability

The datasets supporting the conclusions of this article are available in the NCBI repository. SRR767660 in https://www.ncbi.nlm.nih.gov/sra/?term=SRR767660; SRR768128 in https://www.ncbi.nlm.nih.gov/sra/?term= SRR768128; SRR768129 in https://www.ncbi.nlm.nih.gov/sra/?term= SRR768129; SRR768130 in https://www.ncbi.nlm.nih.gov/sra/?term= SRR768130; SRR768131 in https://www.ncbi.nlm.nih.gov/sra/?term= SRR768131;SRR542478 in https://www.ncbi.nlm.nih.gov/sra/?term= SRR542478; SRR542479 in https://www.ncbi.nlm.nih.gov/sra/?term= SRR542479; SRR542480 in https://www.ncbi.nlm.nih.gov/sra/?term= SRR542480; SRR542481 in https://www.ncbi.nlm.nih.gov/sra/?term= SRR542481; SRR542482 in https://www.ncbi.nlm.nih.gov/sra/?term= SRR542482;SRR654690 in https://www.ncbi.nlm.nih.gov/sra/?term= SRR654690; SRR654692 in https://www.ncbi.nlm.nih.gov/sra/?term= SRR654692; SRR654693 in https://www.ncbi.nlm.nih.gov/sra/?term= SRR654693; SRR654695 in https://www.ncbi.nlm.nih.gov/sra/?term= SRR654695; SRR654699 in https://www.ncbi.nlm.nih.gov/sra/?term= SRR654699; SRR654700 in https://www.ncbi.nlm.nih.gov/sra/?term= SRR654700;SRR531862 in https://www.ncbi.nlm.nih.gov/sra/?term= SRR531862; SRR531863 in https://www.ncbi.nlm.nih.gov/sra/?term= SRR531863; SRR531864 in https://www.ncbi.nlm.nih.gov/sra/?term= SRR531864; SRR531865 in https://www.ncbi.nlm.nih.gov/sra/?term= SRR531865. Not applicable.
