# Supplementary material for: Genome-wide identification and comparative evolutionary analysis of sorbitol metabolism pathway genes in four Rosaceae species and three model plants
Source: BMC Plant Biol. 2022 Jul 15;22:341. doi: 10.1186/s12870-022-03729-z (PMC9284748; doi:10.1186/s12870-022-03729-z)
Supplement: Supplementary file 1 — Additional file 1: Supplementary Table 1. Query sequences of S6PDH, SDH and SOT. Supplementary Table 2. List of identified genes for S6PDH, SDH, and SOT gene families. Supplementary Table 3. Information of identified genes for S6PDH, SDH, and SOT gene families. Supplementary Table 4. Optimal codons for seven species. [file 12870_2022_3729_MOESM1_ESM.doc]

**Supplemental Data**

**Supplementary Table 1.** Query sequences of S6PDH, SDH and SOT.

| Gene name | Entry | Organism | Corresponding locus | Protein length |
| --- | --- | --- | --- | --- |
| S6PDH | P28475 | *Malus domestica* | MDP0000408705 | 310 |
| NAD^+^-SDH | Q9FJ95 | *Arabidopsis thaliana* | AT5G51970.1 | 364 |
| SOT | Q84RI1 | *Malus domestica* | MDP0000688348 | 454 |

Note: Query sequenc retrieved from Uniprot database.

**Supplementary Table 2**. List of identified genes for S6PDH, SDH, and SOT gene families.

Note:

COG: cluster of orthologous group. Genes belong to multiple COGs were labeled in red.

| Species | Gene family | Gene ID | Protein length | COG ID | Chromosome position |
| --- | --- | --- | --- | --- | --- |
| *Arabidopsis* | S6PDH | AT2G21250.1 | 310 | CLS00493 | Chr2:9103207-9105187 |
| *Arabidopsis* | S6PDH | AT2G21260.1 | 310 | CLS00493 | Chr2:9105583-9107380 |
| Apple | S6PDH | **MDP0000220005** | 273 | CLS00493 | chr7:4304303-4306801 |
| Apple | S6PDH | **MDP0000242322** | 316 | CLS00493 | chr7:4305989-4309633 |
| Apple | S6PDH | **MDP0000312001** | 282 | CLS00493 | chr2:30333334-30336381 |
| Pear | S6PDH | **Pbr023248.1** | 311 | CLS00493 | Chr5:5670202-5673068 |
| Pear | S6PDH | **Pbr042781.1** | 322 | CLS00493 | Chr5:5658673-5662343 |
| Mei | S6PDH | Pm017042 | 323 | CLS00493 | Pm5:7297100-7301229 |
| Poplar | S6PDH | Potri.009G125100.1 | 310 | CLS00493 | Chr09:10390387-10392933 |
| Peach | S6PDH | ppa009027m | 310 | CLS00493 | scaffold_2:6008190-6012659 |
| Tomato | S6PDH | Solyc01g110450.2.1 | 310 | CLS00493 | SL2.40ch01:88910585-88913966 |
| Apple | S6PDH | MDP0000133306 | 336 | CLS16328 | unanchored:7139721-7143396 |
| Apple | S6PDH | MDP0000251531 | 310 | CLS16328 | unanchored:7133217-7136207 |
| Apple | S6PDH | MDP0000361351 | 310 | CLS16328 | chr10:11175636-11179027 |
| Apple | S6PDH | MDP0000408705 | 311 | CLS16328 | chr10:11163262-11166655 |
| Apple | S6PDH | MDP0000639894 | 267 | CLS16328 | chr10:11180606-11182413 |
| Apple | S6PDH | MDP0000818877 | 274 | CLS16328 | chr10:11180525-11181987 |
| Pear | S6PDH | **Pbr023248.1** | 311 | CLS16328 | Chr5:5670202-5673068 |
| Pear | S6PDH | **Pbr042781.1** | 322 | CLS16328 | Chr5:5658673-5662343 |
| Mei | S6PDH | Pm021672 | 322 | CLS16328 | Pm6:10493482-10495619 |
| Peach | S6PDH | ppa009007m | 311 | CLS16328 | scaffold_8:10925243-10927698 |
| Apple | S6PDH | **MDP0000220005** | 273 | CLS23028 | chr7:4304303-4306801 |
| Apple | S6PDH | **MDP0000242322** | 316 | CLS23028 | chr7:4305989-4309633 |
| Apple | S6PDH | **MDP0000312001** | 282 | CLS23028 | chr2:30333334-30336381 |
| Pear | S6PDH | Pbr024722.1 | 361 | CLS23028 | Chr2:8253620-8257355 |
| *Arabidopsis* | SDH | AT5G51970.1 | 365 | CLS13131 | Chr5:21111445-21113403 |
| Apple | SDH | MDP0000123910 | 358 | CLS13131 | chr1:25087036-25088743 |
| Apple | SDH | MDP0000149907 | 359 | CLS13131 | unanchored:21356345-21357642 |
| Apple | SDH | MDP0000167088 | 368 | CLS13131 | chr7:23405354-23406795 |
| Apple | SDH | MDP0000171573 | 358 | CLS13131 | chr7:23281847-23283529 |
| Apple | SDH | MDP0000188052 | 368 | CLS13131 | chr7:23301490-23302735 |
| Apple | SDH | MDP0000188054 | 349 | CLS13131 | chr7:23310942-23312187 |
| Apple | SDH | MDP0000250546 | 368 | CLS13131 | chr1:25173127-25174375 |
| Apple | SDH | MDP0000515106 | 369 | CLS13131 | chr1:25177288-25178612 |
| Apple | SDH | MDP0000638442 | 369 | CLS13131 | chr1:25149134-25150444 |
| Apple | SDH | MDP0000707567 | 369 | CLS13131 | chr1:25180931-25182241 |
| Apple | SDH | MDP0000759646 | 389 | CLS13131 | chr14:24043122-24044360 |
| Apple | SDH | MDP0000786110 | 366 | CLS13131 | chr1:25191824-25193641 |
| Apple | SDH | MDP0000807470 | 367 | CLS13131 | chr7:23390960-23392683 |
| Apple | SDH | MDP0000873573 | 369 | CLS13131 | chr1:25182502-25183812 |
| Apple | SDH | MDP0000874667 | 369 | CLS13131 | chr1:25157544-25158783 |
| Pear | SDH | Pbr013912.1 | 379 | CLS13131 | Chr7:13019323-13021076 |
| Pear | SDH | Pbr013913.1 | 369 | CLS13131 | Chr7:13031590-13033474 |
| Pear | SDH | Pbr013914.1 | 369 | CLS13131 | Chr7:13038146-13039397 |
| Pear | SDH | Pbr013915.1 | 369 | CLS13131 | Chr7:13042951-13045433 |
| Pear | SDH | Pbr013916.1 | 369 | CLS13131 | Chr7:13051934-13053377 |
| Pear | SDH | Pbr013917.1 | 367 | CLS13131 | Chr7:13057012-13059031 |
| Pear | SDH | Pbr032772.1 | 369 | CLS13131 | Chr1:8257062-8259836 |
| Pear | SDH | Pbr032773.1 | 369 | CLS13131 | Chr1:8251686-8253003 |
| Pear | SDH | Pbr032774.1 | 369 | CLS13131 | Chr1:8243994-8245311 |
| Pear | SDH | Pbr032775.1 | 369 | CLS13131 | Chr1:8235475-8237204 |
| Pear | SDH | Pbr032776.1 | 369 | CLS13131 | Chr1:8229073-8230308 |
| Pear | SDH | Pbr032777.1 | 369 | CLS13131 | Chr1:8223256-8224960 |
| Pear | SDH | Pbr032778.1 | 359 | CLS13131 | Chr1:8213954-8215641 |
| Mei | SDH | Pm019393 | 367 | CLS13131 | Pm5:23673441-23675177 |
| Poplar | SDH | Potri.012G134900.1 | 365 | CLS13131 | Chr12:15138718-15142158 |
| Peach | SDH | ppa007458m | 368 | CLS13131 | scaffold_2:24766424-24768515 |
| Tomato | SDH | Solyc01g006510.2.1 | 356 | CLS13131 | SL2.40ch01:1093865-1098227 |
| *Arabidopsis* | SOT | AT3G18830.1 | 540 | CLS10657 | Chr3:6488862-6491273 |
| Apple | SOT | MDP0000141961 | 500 | CLS10657 | chr3:15693441-15697343 |
| Apple | SOT | MDP0000197939 | 527 | CLS10657 | chr3:30232275-30234881 |
| Apple | SOT | MDP0000250395 | 445 | CLS10657 | chr12:8951900-8954413 |
| Apple | SOT | MDP0000276801 | 473 | CLS10657 | chr17:20874675-20876932 |
| Apple | SOT | MDP0000285032 | 613 | CLS10657 | chr12:8925509-8929669 |
| Apple | SOT | MDP0000311500 | 809 | CLS10657 | chr12:9260636-9265823 |
| Apple | SOT | MDP0000595941 | 521 | CLS10657 | chr12:9170299-9172080 |
| Apple | SOT | MDP0000596275 | 519 | CLS10657 | chr17:13999472-14001832 |
| Apple | SOT | MDP0000682530 | 521 | CLS10657 | chr12:9066928-9069554 |
| Apple | SOT | MDP0000688348 | 645 | CLS10657 | chr12:8953562-8962152 |
| Apple | SOT | MDP0000688376 | 455 | CLS10657 | chr12:8996955-8999599 |
| Apple | SOT | MDP0000787701 | 600 | CLS10657 | chr12:8847651-8850448 |
| Apple | SOT | MDP0000896307 | 527 | CLS10657 | chr12:2414264-2416493 |
| Apple | SOT | MDP0000940086 | 539 | CLS10657 | chr1:8266573-8269190 |
| Pear | SOT | Pbr018463.1 | 536 | CLS10657 | scaffold277.0:457323-460601 |
| Pear | SOT | Pbr018464.1 | 538 | CLS10657 | scaffold277.0:482451-485638 |
| Pear | SOT | Pbr018903.1 | 521 | CLS10657 | Chr7:13878110-13880499 |
| Pear | SOT | Pbr018904.1 | 456 | CLS10657 | Chr7:13914274-13916559 |
| Pear | SOT | Pbr018906.1 | 529 | CLS10657 | Chr7:13929491-13932144 |
| Pear | SOT | Pbr018908.1 | 527 | CLS10657 | Chr7:13962795-13965291 |
| Pear | SOT | Pbr018910.1 | 527 | CLS10657 | Chr7:14036776-14039272 |
| Pear | SOT | Pbr019072.1 | 563 | CLS10657 | Chr8:15137413-15140247 |
| Pear | SOT | Pbr019074.1 | 474 | CLS10657 | Chr8:15120720-15123012 |
| Pear | SOT | Pbr019075.1 | 440 | CLS10657 | Chr8:15111822-15114175 |
| Pear | SOT | Pbr022830.1 | 529 | CLS10657 | Chr3:1812296-1814945 |
| Pear | SOT | Pbr034135.1 | 474 | CLS10657 | scaffold637.0:109119-111460 |
| Pear | SOT | Pbr034137.1 | 528 | CLS10657 | scaffold637.0:120806-123457 |
| Pear | SOT | Pbr034138.1 | 486 | CLS10657 | scaffold637.0:129943-132271 |
| Pear | SOT | Pbr037511.1 | 482 | CLS10657 | scaffold764.0:47023-49531 |
| Pear | SOT | Pbr037512.1 | 538 | CLS10657 | scaffold764.0:74877-77995 |
| Pear | SOT | Pbr037515.1 | 527 | CLS10657 | scaffold764.0:150392-152568 |
| Pear | SOT | Pbr038546.1 | 528 | CLS10657 | Chr5:8197570-8201837 |
| Pear | SOT | Pbr038547.1 | 527 | CLS10657 | Chr5:8227701-8230052 |
| Pear | SOT | Pbr038549.1 | 539 | CLS10657 | Chr5:8307294-8309941 |
| Pear | SOT | Pbr040466.1 | 527 | CLS10657 | Chr3:14387203-14389811 |
| Mei | SOT | Pm021605 | 528 | CLS10657 | Pm6:9921176-9923338 |
| Mei | SOT | Pm021606 | 528 | CLS10657 | Pm6:9934512-9936674 |
| Mei | SOT | Pm021647 | 538 | CLS10657 | Pm6:10252824-10255161 |
| Mei | SOT | Pm021649 | 531 | CLS10657 | Pm6:10299770-10301738 |
| Mei | SOT | Pm021715 | 593 | CLS10657 | Pm6:10817390-10820176 |
| Mei | SOT | Pm030070 | 671 | CLS10657 | scaffold438:43996-46853 |
| Mei | SOT | Pm031233 | 511 | CLS10657 | scaffold877:6743-8641 |
| Poplar | SOT | Potri.004G152300.1 | 532 | CLS10657 | Chr04:17416629-17419361 |
| Poplar | SOT | Potri.009G113600.1 | 534 | CLS10657 | Chr09:9660055-9663154 |
| Peach | SOT | **ppa004456m** | 510 | CLS10657 | scaffold_8:12530334-12533494 |
| Peach | SOT | ppa014975m | 528 | CLS10657 | scaffold_8:12901101-12903265 |
| Peach | SOT | ppa015090m | 528 | CLS10657 | scaffold_8:12873747-12875895 |
| Peach | SOT | ppa015294m | 539 | CLS10657 | scaffold_8:12519658-12521978 |
| Peach | SOT | ppa018884m | 528 | CLS10657 | scaffold_8:12913802-12915970 |
| Peach | SOT | ppa021469m | 491 | CLS10657 | scaffold_8:12481392-12483430 |
| Peach | SOT | ppa021762m | 529 | CLS10657 | scaffold_8:12442088-12444233 |
| Peach | SOT | ppa024323m | 535 | CLS10657 | scaffold_8:12954834-12957297 |
| Tomato | SOT | Solyc01g109460.2.1 | 542 | CLS10657 | SL2.40ch01:88167522-88170694 |
| Apple | SOT | **MDP0000296050** | 655 | CLS25070 | chr12:8892824-8898783 |
| Pear | SOT | Pbr018465.1 | 492 | CLS25070 | scaffold277.0:536975-540403 |
| Pear | SOT | Pbr037514.1 | 480 | CLS25070 | scaffold764.0:131081-133924 |
| Pear | SOT | Pbr038548.1 | 480 | CLS25070 | Chr5:8247534-8251889 |
| Apple | SOT | **MDP0000296050** | 655 | CLS25071 | chr12:8892824-8898783 |
| Peach | SOT | **ppa004456m** | 510 | CLS25071 | scaffold_8:12530334-12533494 |
| Mei | SOT | Pm021648 | 509 | CLS28811 | Pm6:10283244-10285874 |
| Peach | SOT | **ppa004456m** | 510 | CLS28811 | scaffold_8:12530334-12533494 |

**Supplementary Table 3.** Information of identified genes for S6PDH, SDH, and SOT gene families.

Note:

Frequency of optimal codons (FOP), effective number of codons, rate of GC content, and rate of GC3 content

| Gene_family | GeneID | Gene duplication type | FOP | ENC | GC | GC3 |
| --- | --- | --- | --- | --- | --- | --- |
| S6PDH | AT2G21250.1 | Tandem duplication | 0.36 | 59.85 | 0.47 | 0.5 |
| S6PDH | AT2G21260.1 | Tandem duplication | 0.38 | 57.93 | 0.46 | 0.47 |
| S6PDH | Potri.009G125100.1 | Dispersed duplication | 0.43 | 57.68 | 0.45 | 0.44 |
| S6PDH | Solyc01g110450.2.1 | Singleton | 0.36 | 55.79 | 0.46 | 0.44 |
| S6PDH | MDP0000133306 | Dispersed duplication | 0.43 | 54.64 | 0.46 | 0.47 |
| S6PDH | MDP0000220005 | Dispersed duplication | 0.46 | 58.19 | 0.45 | 0.41 |
| S6PDH | MDP0000242322 | Dispersed duplication | 0.44 | 58.47 | 0.45 | 0.42 |
| S6PDH | MDP0000251531 | Dispersed duplication | 0.42 | 57.98 | 0.46 | 0.49 |
| S6PDH | MDP0000312001 | Dispersed duplication | 0.45 | 59.69 | 0.47 | 0.46 |
| S6PDH | MDP0000361351 | Dispersed duplication | 0.43 | 55.93 | 0.47 | 0.5 |
| S6PDH | MDP0000408705 | Dispersed duplication | 0.43 | 56.16 | 0.47 | 0.5 |
| S6PDH | MDP0000639894 | Dispersed duplication | 0.43 | 56.15 | 0.46 | 0.48 |
| S6PDH | MDP0000818877 | Dispersed duplication | 0.43 | 56.35 | 0.45 | 0.47 |
| S6PDH | Pbr023248.1 | Proximal duplication | 0.41 | 57.2 | 0.47 | 0.48 |
| S6PDH | Pbr024722.1 | Proximal duplication | 0.41 | 57.97 | 0.47 | 0.46 |
| S6PDH | Pbr042781.1 | Proximal duplication | 0.41 | 56.67 | 0.46 | 0.48 |
| S6PDH | Pm017042 | Proximal duplication | 0.43 | 57.65 | 0.44 | 0.4 |
| S6PDH | Pm021672 | Dispersed duplication | 0.36 | 54.97 | 0.46 | 0.5 |
| S6PDH | ppa009007m | Dispersed duplication | 0.34 | 54.66 | 0.46 | 0.5 |
| S6PDH | ppa009027m | Dispersed duplication | 0.42 | 57.42 | 0.44 | 0.4 |
| SDH | AT5G51970.1 | Dispersed duplication | 0.46 | 51.43 | 0.46 | 0.35 |
| SDH | Potri.012G134900.1 | Tandem duplication | 0.39 | 52.53 | 0.47 | 0.38 |
| SDH | Solyc01g006510.2.1 | Singleton | 0.43 | 49.23 | 0.46 | 0.33 |
| SDH | MDP0000123910 | Dispersed duplication | 0.41 | 56.62 | 0.51 | 0.5 |
| SDH | MDP0000149907 | Dispersed duplication | 0.42 | 53.87 | 0.51 | 0.49 |
| SDH | MDP0000167088 | Tandem duplication | 0.41 | 58.59 | 0.51 | 0.49 |
| SDH | MDP0000171573 | Dispersed duplication | 0.39 | 57.51 | 0.51 | 0.49 |
| SDH | MDP0000188052 | Proximal duplication | 0.4 | 58.38 | 0.51 | 0.49 |
| SDH | MDP0000188054 | Proximal duplication | 0.39 | 58.6 | 0.49 | 0.47 |
| SDH | MDP0000250546 | Proximal duplication | 0.39 | 57.36 | 0.51 | 0.51 |
| SDH | MDP0000515106 | Tandem duplication | 0.43 | 55.35 | 0.5 | 0.49 |
| SDH | MDP0000638442 | Dispersed duplication | 0.42 | 55.35 | 0.5 | 0.48 |
| SDH | MDP0000707567 | Dispersed duplication | 0.42 | 55.35 | 0.5 | 0.48 |
| SDH | MDP0000759646 | Dispersed duplication | 0.4 | 56.41 | 0.5 | 0.5 |
| SDH | MDP0000786110 | Dispersed duplication | 0.37 | 57.14 | 0.51 | 0.53 |
| SDH | MDP0000807470 | Tandem duplication | 0.42 | 58.61 | 0.51 | 0.5 |
| SDH | MDP0000873573 | Tandem duplication | 0.42 | 57.81 | 0.51 | 0.48 |
| SDH | MDP0000874667 | Proximal duplication | 0.39 | 57.34 | 0.52 | 0.52 |
| SDH | Pbr013912.1 | WGD/segmental duplication | 0.39 | 56.22 | 0.5 | 0.49 |
| SDH | Pbr013913.1 | WGD/segmental duplication | 0.4 | 57.32 | 0.51 | 0.49 |
| SDH | Pbr013914.1 | Tandem duplication | 0.4 | 57.82 | 0.51 | 0.48 |
| SDH | Pbr013915.1 | Proximal duplication | 0.37 | 57.7 | 0.52 | 0.51 |
| SDH | Pbr013916.1 | WGD/segmental duplication | 0.41 | 58.86 | 0.51 | 0.49 |
| SDH | Pbr013917.1 | Tandem duplication | 0.43 | 56.4 | 0.51 | 0.51 |
| SDH | Pbr032772.1 | WGD/segmental duplication | 0.41 | 58.62 | 0.51 | 0.48 |
| SDH | Pbr032773.1 | Tandem duplication | 0.43 | 55.86 | 0.5 | 0.49 |
| SDH | Pbr032774.1 | Tandem duplication | 0.42 | 55.49 | 0.5 | 0.48 |
| SDH | Pbr032775.1 | Tandem duplication | 0.37 | 56.89 | 0.51 | 0.5 |
| SDH | Pbr032776.1 | Tandem duplication | 0.38 | 58.13 | 0.51 | 0.5 |
| SDH | Pbr032777.1 | WGD/segmental duplication | 0.37 | 57.91 | 0.51 | 0.51 |
| SDH | Pbr032778.1 | WGD/segmental duplication | 0.39 | 56.95 | 0.51 | 0.49 |
| SDH | Pm019393 | Dispersed duplication | 0.35 | 60.15 | 0.53 | 0.54 |
| SDH | ppa007458m | Dispersed duplication | 0.33 | 59.07 | 0.53 | 0.54 |
| SOT | AT3G18830.1 | Dispersed duplication | 0.31 | 55.85 | 0.5 | 0.49 |
| SOT | Potri.004G152300.1 | WGD/segmental duplication | 0.38 | 55.74 | 0.45 | 0.36 |
| SOT | Potri.009G113600.1 | WGD/segmental duplication | 0.4 | 55.84 | 0.44 | 0.35 |
| SOT | Solyc01g109460.2.1 | Singleton | 0.36 | 54.23 | 0.47 | 0.45 |
| SOT | MDP0000141961 | Dispersed duplication | 0.36 | 57.89 | 0.49 | 0.51 |
| SOT | MDP0000197939 | Dispersed duplication | 0.38 | 56.89 | 0.48 | 0.5 |
| SOT | MDP0000250395 | Dispersed duplication | 0.36 | 58.2 | 0.49 | 0.52 |
| SOT | MDP0000276801 | Dispersed duplication | 0.36 | 56.32 | 0.49 | 0.53 |
| SOT | MDP0000285032 | Dispersed duplication | 0.36 | 57.31 | 0.49 | 0.52 |
| SOT | MDP0000296050 | Dispersed duplication | 0.33 | 56.2 | 0.52 | 0.55 |
| SOT | MDP0000311500 | Dispersed duplication | 0.36 | 57.72 | 0.5 | 0.55 |
| SOT | MDP0000595941 | Dispersed duplication | 0.34 | 55.56 | 0.51 | 0.57 |
| SOT | MDP0000596275 | Dispersed duplication | 0.35 | 57.79 | 0.51 | 0.53 |
| SOT | MDP0000682530 | Dispersed duplication | 0.38 | 56.91 | 0.48 | 0.51 |
| SOT | MDP0000688348 | Dispersed duplication | 0.36 | 56.61 | 0.48 | 0.49 |
| SOT | MDP0000688376 | Dispersed duplication | 0.38 | 58.96 | 0.49 | 0.52 |
| SOT | MDP0000787701 | Dispersed duplication | 0.32 | 57.45 | 0.51 | 0.55 |
| SOT | MDP0000896307 | Dispersed duplication | 0.36 | 58.13 | 0.51 | 0.54 |
| SOT | MDP0000940086 | Dispersed duplication | 0.32 | 56.24 | 0.52 | 0.56 |
| SOT | Pbr018463.1 | Tandem duplication | 0.27 | 54.67 | 0.53 | 0.63 |
| SOT | Pbr018464.1 | Tandem duplication | 0.3 | 57.24 | 0.52 | 0.57 |
| SOT | Pbr018465.1 | Tandem duplication | 0.3 | 57.06 | 0.52 | 0.55 |
| SOT | Pbr018903.1 | Proximal duplication | 0.32 | 56.69 | 0.5 | 0.56 |
| SOT | Pbr018904.1 | Dispersed duplication | 0.32 | 52.85 | 0.47 | 0.5 |
| SOT | Pbr018906.1 | Dispersed duplication | 0.34 | 56.98 | 0.48 | 0.49 |
| SOT | Pbr018908.1 | Proximal duplication | 0.32 | 58.99 | 0.48 | 0.5 |
| SOT | Pbr018910.1 | Proximal duplication | 0.32 | 58.99 | 0.48 | 0.5 |
| SOT | Pbr019072.1 | Dispersed duplication | 0.32 | 55.23 | 0.48 | 0.48 |
| SOT | Pbr019074.1 | Tandem duplication | 0.33 | 57.11 | 0.48 | 0.49 |
| SOT | Pbr019075.1 | Tandem duplication | 0.34 | 56.29 | 0.48 | 0.46 |
| SOT | Pbr022830.1 | Dispersed duplication | 0.34 | 58.8 | 0.47 | 0.49 |
| SOT | Pbr034135.1 | Proximal duplication | 0.31 | 56.93 | 0.48 | 0.49 |
| SOT | Pbr034137.1 | Tandem duplication | 0.35 | 57.1 | 0.47 | 0.47 |
| SOT | Pbr034138.1 | Tandem duplication | 0.33 | 57.58 | 0.47 | 0.47 |
| SOT | Pbr037511.1 | Tandem duplication | 0.23 | 53.11 | 0.54 | 0.65 |
| SOT | Pbr037512.1 | Tandem duplication | 0.3 | 57.43 | 0.52 | 0.57 |
| SOT | Pbr037514.1 | Proximal duplication | 0.3 | 57.74 | 0.52 | 0.56 |
| SOT | Pbr037515.1 | Dispersed duplication | 0.31 | 56.96 | 0.5 | 0.54 |
| SOT | Pbr038546.1 | Dispersed duplication | 0.32 | 58.72 | 0.49 | 0.51 |
| SOT | Pbr038547.1 | Dispersed duplication | 0.32 | 55.81 | 0.51 | 0.55 |
| SOT | Pbr038548.1 | Dispersed duplication | 0.3 | 56.74 | 0.52 | 0.57 |
| SOT | Pbr038549.1 | Dispersed duplication | 0.31 | 56.47 | 0.52 | 0.57 |
| SOT | Pbr040466.1 | Dispersed duplication | 0.31 | 56.57 | 0.51 | 0.54 |
| SOT | Pm021605 | Tandem duplication | 0.3 | 54.43 | 0.52 | 0.58 |
| SOT | Pm021606 | Tandem duplication | 0.3 | 54.43 | 0.52 | 0.58 |
| SOT | Pm021647 | Tandem duplication | 0.28 | 55.57 | 0.53 | 0.6 |
| SOT | Pm021648 | WGD/segmental duplication | 0.27 | 54.15 | 0.54 | 0.64 |
| SOT | Pm021649 | Tandem duplication | 0.3 | 55.41 | 0.51 | 0.57 |
| SOT | Pm021715 | Dispersed duplication | 0.27 | 52.65 | 0.54 | 0.65 |
| SOT | Pm030070 | Dispersed duplication | 0.25 | 52.66 | 0.54 | 0.63 |
| SOT | Pm031233 | Dispersed duplication | 0.26 | 58.08 | 0.54 | 0.66 |
| SOT | ppa004456m | Tandem duplication | 0.27 | 53.45 | 0.54 | 0.64 |
| SOT | ppa014975m | Tandem duplication | 0.29 | 55.76 | 0.52 | 0.59 |
| SOT | ppa015090m | Proximal duplication | 0.3 | 55.52 | 0.52 | 0.59 |
| SOT | ppa015294m | Tandem duplication | 0.32 | 55.67 | 0.5 | 0.56 |
| SOT | ppa018884m | Tandem duplication | 0.29 | 55.73 | 0.52 | 0.59 |
| SOT | ppa021469m | Proximal duplication | 0.25 | 51.4 | 0.55 | 0.67 |
| SOT | ppa021762m | WGD/segmental duplication | 0.26 | 52.34 | 0.53 | 0.65 |
| SOT | ppa024323m | Proximal duplication | 0.3 | 56.19 | 0.52 | 0.58 |

**Supplementary Table 4.** Optimal codons for seven species.

| Amino acid | Tomato | Poplar | *Arabidopsis* | Peach | Mei | Apple | Pear |
| --- | --- | --- | --- | --- | --- | --- | --- |
| Ala | GCA | GCT | GCT | GCT | GCT | GCT | GCT |
| Arg | AGA | AGA | AGA | AGA | AGA | AGG | AGG |
| Asn | AAT | AAT | AAT | AAT | AAT | AAT | AAT |
| Asp | GAT | GAT | GAT | GAT | GAT | GAT | GAT |
| Cys | TGT | TGT | TGT | TGT | TGT | TGT | TGT |
| Gln | CAA | CAA | CAA | CAA | CAA | CAA | CAG |
| Glu | GAA | GAA | GAA | GAA | GAA | GAG | GAG |
| Gly | GGA | GGT | GGT | GGT | GGT | GGT | GGT |
| His | CAT | CAT | CAT | CAT | CAT | CAT | CAT |
| Ile | ATT | ATT | ATT | ATT | ATT | ATT | ATT |
| Leu | TTA | TTG | CTT | TTG | TTG | None | None |
| Lys | AAA | AAG | AAG | AAA | AAG | AAG | AAG |
| Phe | TTT | TTT | TTT | TTT | TTT | TTT | TTT |
| Pro | CCA | CCT | CCT | CCA | CCA | CCT | CCT |
| Ser | TCA | TCT | TCT | TCT | TCT | TCT | TCT |
| Thr | ACA | ACT | ACT | ACA | ACT | ACT | ACT |
| Tyr | TAT | TAT | TAT | TAT | TAT | TAT | TAT |
| Val | GTT | GTT | GTT | GTT | GTT | GTT | GTG |
